# Immobilization of Inorganic Phosphorus on Soils by Zinc Oxide Engineered Nanoparticles

**DOI:** 10.3390/toxics13050363

**Published:** 2025-04-30

**Authors:** Jonathan Suazo-Hernández, Rawan Mlih, Marion Bustamante, Carmen Castro-Castillo, María de la Luz Mora, María de los Ángeles Sepúlveda-Parada, Catalina Mella, Pablo Cornejo, Antonieta Ruiz

**Affiliations:** 1 Center of Plant, Soil Interaction and Natural Resources Biotechnology, Scientific and Biotechnological Bioresource Nucleus (BIOREN-UFRO), Universidad de La Frontera, Avenida Francisco Salazar, Temuco 01145, Chile; mariluz.mora@ufrontera.cl; 2 Department of Chemical Sciences and Natural Resources, Universidad de La Frontera, Avenida Francisco Salazar, P.O. Box 54-D, Temuco 01145, Chile; 3 Institute of Bio- and Geosciences, Agrosphere (IBG-3), Forschungszentrum Juelich (FZJ), 52425 Juelich, Germany; r.mlih@fz-juelich.de; 4 Institute of Water and Environment (IWE), Al Azhar University-Gaza, Gaza P.O. Box 1277, Palestine; 5 Doctoral Program in Engineering at the MacroFacultad de Ingeniería UFRO-UBB-UTAL, Temuco 4780000, Chile; marionbustamante.v@gmail.com; 6 LabMAM, Department of Chemical Engineering, Biotechnology and Materials, FCFM, Universidad de Chile, Santiago 8370456, Chile; carmen.castro.c@uchile.cl; 7 Spectroscopy Laboratory (Vis-IF) and Sustainable Soil Management, Department of Soil Science and Natural Resources, Faculty of Agronomy, Universidad de Concepción, Vicente Méndez 595, Casilla 537, Chillán 3812120, Chile; angeles.sepulvedap@gmail.com; 8 Doctorado en Ciencias de Recursos Naturales, Universidad de La Frontera, Temuco 4811230, Chile; c.mella10@ufromail.cl; 9 Plant Stress Physiology Laboratory, Centro de Estudios Avanzados en Fruticultura (CEAF), Rengo 2940000, Chile; pablo.cornejo@pucv.cl; 10 Centro Tecnológico de Suelos y Cultivos (CTSyC), Facultad de Ciencias Agrarias, Universidad de Talca, Talca 3460000, Chile

**Keywords:** engineered nanoparticles, zinc oxide nanoparticles, inorganic phosphate, eutrophication, adsorption, Ultisol, Mollisol

## Abstract

The overuse of inorganic phosphate fertilizers in soils has led to the transfer of inorganic phosphorus (Pi) to aquatic ecosystems, resulting in eutrophication. Adsorption–desorption studies in batch systems were used to evaluate the effect of adding 1% zinc oxide (ZnO) engineered nanoparticles (ENPs) on Pi retention in Ultisol, and Mollisol soils. The 1% ZnO–ENPs showed increased chemical properties such as pH, electrical conductivity, and organic matter content, and reduce nutrient bioavailability (P, N, and Zn), and physical properties such as surface area and pore size of the two soils. The kinetic data of Pi adsorption on Ultisol, Mollisol, Ultisol + 1% ZnO–ENP, and Mollisol + 1% ZnO–ENP systems fitted well to the pseudo-second-order model (r^2^ ≥ 0.942, and χ^2^ ≤ 61), and the Elovich model (r^2^ ≥ 0.951, and χ^2^ ≤ 32). Pi adsorption isotherms for the Ultisol soil adequately fitted to the Freundlich model (r^2^ = 0.976, and χ^2^ = 16), and for the Mollisol soil, the Langmuir model (r^2^ = 0.991, and χ^2^ = 3) had a better fit to the data. With 1% ZnO–ENPs, the linear, Langmuir, and Freundlich models correctly described the Pi adsorption data. Pi desorption was reduced in the Ultisol compared to the Mollisol soil, and with 1% ZnO–ENPs further decreased Pi desorption in both soils. Therefore, ENPs can be used as a new alternative material for Pi fixation in agricultural soils and contribute to mitigating eutrophication issues of aqueous systems.

## 1. Introduction

Inorganic phosphorus (Pi) is a crucial element for optimal plant growth. However, most of the world’s agricultural soils lack this nutrient [1]. To address the lack of Pi, farmers often add large amounts of organic amendments (i.e., straw, manure, compost, and municipal solid waste) together with phosphate fertilizers (i.e., triple superphosphate, single superphosphate, and dicalcium phosphate) [2,3]. It is estimated that around 24 million tons of Pi are incorporated into agricultural soils annually [4], and according to estimations models, it will double by 2050 [5]. This phenomenon poses a significant risk to the environment and human health due to the excessive release of Pi, which causes eutrophication (i.e., an overabundance of harmful algae and plants) in water bodies, particularly reservoirs and lakes, leading to deterioration of their water quality. Eutrophication can cause aquatic ecosystems to collapse and result in a major economic loss for the region [6,7]. Therefore, there is a serious need to explore new technologies to prevent Pi leaching into freshwater bodies and improve its retention/fixation capacity onto agricultural soils that have a low affinity for this nutrient [8].

Previous studies have demonstrated that adding specific compounds to the soil can increase Pi immobilization and prevent its leaching into the environment. Among these compounds are organic amendments, industrial by-products such as iron-modified calcite, attapulgite, bentonite, and dolomite [9], biochar [10] and biochar-polyacrylamide composite [11], red sludge [12], aluminum-based compounds [13], and many others. However, many of these substrates suffer from poor Pi fixation efficiency and are economically unfeasible. Additionally, these substrates contribute to the leaching of significant amounts of heavy metals into the groundwater [14].

In recent years, nanotechnology has emerged as one of the fastest-growing technology sectors worldwide [15]. The nanotechnology market in 2020 was about USD 3.78 billion, and it is projected to reach USD 74.1 billion by 2032 [16]. Nowadays, engineered nanoparticles (ENPs) are being used extensively in the fields of energy, food, agriculture, transportation, and health, helping to improve the quality of human life [17,18]. Consequently, the application of nanotechnology in soil remediation with reactive ENPs has garnered significant interest [19]. In this sense, the use of ENPs with catalytic properties proved to reduce the concentration of persistent organic molecules, such as polycyclic aromatic hydrocarbons (PAHs) [20] and pesticides [21]. On the other hand, ENPs offer huge surface areas which makes them excellent adsorbates for heavy metals such as copper (Cu), zinc (Zn), and cadmium (Cd) [22,23] and metalloids such as arsenic (As) and mercury (Hg) [24]. However, this promising technology has scarcely been studied to control the eutrophication of aquatic systems to prevent Pi transport/mobilization into the soils. To achieve effective immobilization of Pi in agricultural soils using ENPs, it is essential to meet two key criteria: firstly, ENPs must be highly transportable in soils, and secondly, they must possess a high adsorption capacity and affinity for Pi [25]. In particular, metallic oxide ENPs, particularly zinc oxide ENPs (ZnO–ENPs) are characterized by low cost, low aggregation, environmental stability, and non-toxicity at low concentrations for soil organisms [26,27]. Several studies have assessed the interaction between ZnO–ENPs and Pi using model systems, revealing that ZnO–ENPs, upon dissolution, release Zn^2+^ cations into the solution [28,29]. These cations can subsequently form complexes and precipitates with Pi species (dihydrogen phosphate (H_2_PO_4_^−^), hydrogen phosphate (HPO_4_^2−^), and phosphate (PO_4_^3−^)) present in the solution. Pi can also be adsorbed onto the surface of ZnO–ENPs through inner sphere mechanisms due to replacement of hydroxyl groups (–OH) by Pi [30,31].

The aims of this study are to investigate the effect of ZnO–ENPs (i) on the physicochemical properties of two agricultural soils, i.e., Ultisol and Mollisol, and (ii) their role in Pi immobilization in the soils.

## 2. Materials and Methods

### 2.1. Study Materials

Ultisol and Mollisol soils were collected from southern (37°42′54.5″ S 72°37′09.8″ W) and central Chile (33°00′09.6″ S 71°21′09.8″ W), respectively, at a depth of 0–20 cm. Both soils were air-dried, passed through a 2 mm sieve, and then stored at 4 °C for later use. The ZnO–ENP nanopowder < 100 nm (CAS number 1314-13-2) was purchased from Sigma-Aldrich (St. Louis, MO, USA). These ENPs have a surface area ranging from 10 to 25 m^2^ g^−1^ and an isoelectric point of 8.1.

### 2.2. Characterization of Soils Without and with 1% ZnO–ENPs

The physicochemical properties and bioavailability of Zn in the soils were determined at 0 min and after 1440 min of incubation with 1% ZnO–ENPs. The pH of the initial soils was determined using a 1:2.5 soil:water ratio. A 1:5 soil:water ratio was used to measure electrical conductivity (EC) [32]. Inorganic nitrogen (N) in the soil samples was determined with 2 mol L^−1^ potassium chloride (KCl; Sigma-Aldrich, St. Louis, MO, USA) [32]. Available P was analyzed with sodium bicarbonate (0.5 mol L^−1^ NaHCO_3_ at pH 8.50) (Sigma-Aldrich, St. Louis, MO, USA) [33]. The organic matter (OM) content of each soil was measured using 20 mL of potassium dichromate (K_2_Cr_2_O_7_) (Sigma-Aldrich, St. Louis, MO), and sulfuric acid (H_2_SO_4_; Sigma-Aldrich, St. Louis, MO, USA) as oxidizing agents [32]. Cation exchange capacity (CEC) was calculated as the sum of sodium (Na), potassium (K), magnesium (Mg), and calcium (Ca) extracted with ammonium acetate (NH_4_CH_3_CO_2_) at pH = 7.0) (Sigma-Aldrich, St. Louis, MO, USA). Exchangeable aluminum (Al) was extracted with KCl 1 mol L^−1^ and measured by atomic absorption spectroscopy (AAS, iCE 3500, Thermo Fisher Scientific, Waltham, MA, USA). The effective cation exchange capacity (ECEC) was calculated as the sum of exchangeable Al plus the CEC. Available Zn, Cu, and Fe were determined by the diethylenetriaminepentaacetic acid (DTPA) (Sigma-Aldrich, St. Louis, MO, USA) extraction method [34]. To establish the isoelectric point (IEP), the soil samples were suspended in sodium chloride (NaCl; Sigma-Aldrich, St. Louis, MO, USA) and measured using a Zeta Meter ZM–77 (Zeta Meter Inc., New York, NY, USA).

### 2.3. Batch Adsorption Equilibrium Studies

The adsorption studies (kinetic adsorptions, isotherm adsorptions, weight ENPs, and pH) were done with 20 mL of Pi solution (H_2_PO_4_^−^) onto Ultisol, Mollisol, Ultisol + 1% ZnO–ENPs, and Mollisol + 1% ZnO–ENPs at 20 ± 1 °C of 0.01 mol L^−1^ NaCl. The experimental conditions of the Pi adsorption studies are outlined in Table 1.

The suspensions were separated from the solid through centrifugation at 12,000× *g* rpm for 10 min. Afterward, suspensions were filtered using mixed cellulose ester (MCE) membrane filters (0.45 μm pore size). The Pi concentration in the supernatant was measured according to the Murphy and Riley method [35] using a spectrophotometer model Rayleigh UV-2601 (BRAIC Co., Ltd., Beijing, China). The Pi concentration adsorbed (q_e_, mmol kg^−1^) was calculated by Equation (1).

(1) $$q_{e}=\frac{(C_{0}-C_{t})V}{\left(w\right)}$$

where $C_{0}$ (mmol L^−1^) is the initial concentration of Pi, C_t_ (mmol L^−1^) is the concentration of Pi at time t, or the equilibrium concentration, V (L) is the volume, and w is the weight (kg) of soil.

### 2.4. Batch Desorption Studies

To evaluate the effect of ZnO–ENPs on Pi desorption from Ultisol, Mollisol, Ultisol + 1% ZnO–ENP, and Mollisol + 1% ZnO–ENP systems, 20 mL of solution of 6.46 mmol L^−1^ of Pi were added at pH 5.5 ± 0.1 (pH values were fitted by adding HCl and NaOH) at 20 ± 1 °C of 0.01 mol L^−1^ NaCl (ionic strength) in polypropylene tubes. The tubes were shaken at 200 rpm for 1440 min in an orbital shaker. After centrifugation, the supernatant was filtered, and Pi concentration in suspension was measured using a spectrophotometer at the wavelength of 880 nm. Consecutively, 10 mL of a solution of 0.01 mol L^−1^ NaCl (ionic strength) without Pi at pH 5.5 ± 0.1 were replaced by the original solution four times (successively), and for each time, the tubes were shaken under the same previously mentioned condition. Finally, the Pi concentration in suspension was measured as described above.

### 2.5. Data Analysis

Data analysis of Pi adsorption–desorption studies and figures were plotted using the Origin 2019b software (OriginLab Corp., Northampton, MA, USA). Standard solutions and reagent blanks were included with each batch experiment. Adsorption–desorption studies were performed in triplicate. The experimental kinetic data were fitted to the pseudo-first-order (PFO), pseudo-second-order (PSO), and Elovich models. The mathematical equations are provided in Table S1. The experimental data of the Pi adsorption isotherms were fitted to the linear mathematical (Henry’s law), Langmuir, Freundlich, and Temkin models; these equations are given in Table S2. The data obtained from the experimental studies of kinetics and adsorption isotherms were assessed through the coefficient of determination (r^2^) and chi-square (χ^2^) for all models, except for the linear model, where only the r^2^ value was used.

## 3. Results and Discussion

### 3.1. Soil Physicochemical Properties with and Without ZnO–ENPs

The initial physicochemical properties of the soils, such as pH, EC, OM, ECEC, available macro- and micronutrient concentrations, BET surface area, and pore volume, were analyzed before the beginning of the adsorption–desorption experiments (Table 2). The initial soils had acid pH values (6.14 for Ultisol, and 6.33 Mollisol), low EC values ≤ 1 (0.039 ds m^−1^ for Ultisol, and 0.078 ds m^−1^ for Mollisol), and medium values of % OM (2.07% OM for Ultisol and 2.36% OM for Mollisol). In terms of nutrient concentration, the Ultisol soil exhibited a higher concentration of available Fe and Cu than the Mollisol soil. In contrast, the Mollisol soil presented higher values of available P than the Ultisol soil. However, both soils showed similar available N concentrations. Similarly, the Mollisol soil showed a high concentration of Zn (15.3 mg kg^−1^) in contrast to the Ultisol soil, which had a low concentration of available Zn (0.69 mg kg^−1^). This difference may be attributed to the elevated OM % in the Mollisol soil, as humic and fulvic acids which significantly influence Zn^2+^ accumulation [36].

After adding 1% ZnO–ENPs to both soils, the pH of the two soils increased by approximately 2 units. The increase in pH in the Mollisol and Ultisol soils is related to the consumption of H+ as a result of the dissolution of ZnO–ENPs in water (Equation (2)) [37,38]. Similar pH results were reported by Shemawar et al. [39] using 100 mg ZnO–ENP soil kg^−1^ and by Suazo-Hernández et al. [40] using 1% ZnO–ENPs in a volcanic soil.

(2) $$ZnO_{\left(S\right)}+{2H}_{\left(aq\right)}^{+}↔{Zn}_{\left(aq\right)}^{2+}+H_{2}O_{\left(l\right)}$$

Soil EC, which provides the measure of total soluble salts, was drastically affected by the application of 1% ZnO–ENPs, with the most pronounced change in EC noted in the Mollisol soil (0.097 dS m^−1^) compared to the Ultisol (0.054 dS m^−1^) (Table 2). Moreover, soil %OM, an important indicator of soil fertility, increased in both soils, being higher for the Mollisol + 1% ZnO–ENP system (2.68%) than for the Ultisol + 1% ZnO–ENPs (2.13%). The increase in OM values may be attributed to elevated microbial activity resulting from the activation of microorganisms (fungi, bacteria, and actinomycetes) and soil enzymes [41] facilitated by the action of ENPs, which enhances the decomposition of soil OM. However, it is also likely that these results are associated with soil pH variation, as boosting soil pH enhances the solubility of dissolved organic carbon by promoting its desorption from minerals.

The Ultisol soil treated with 1% ZnO–ENPs showed a Zn concentration in DTPA 1262.3 times higher compared to the untreated soil, whereas for the Mollisol soil with 1% ZnO–ENPs, the Zn concentration was 59.61 times higher than the soil without ENPs. ZnO–ENPs typically dissolve rapidly in acidic soils and slowly in neutral and alkaline soils [42]. Therefore, the notable increase in the concentration of available Zn with 1% ZnO–ENPs for both soils and particularly for the Ultisol soil may be due to its more acidic pH compared to the Mollisol soil, which would favor the dissolution of ENPs [43]. Several studies have reported an increase in the concentration of available Zn in soils treated with ZnO–ENPs, which has been much higher in soils with acidic rather than basic pH [44,45,46]. In addition, 1% ZnO–ENPs in both soils caused a decrease in P, N, and Cu bioavailability. Concerning this phenomenon, Shemawar et al. [39], suggested that ZnO–ENPs can cause a reduction in the solubilization process responsible for the release of Pi in solution, which for this study may also be occurring for N, Fe, and Cu. Likewise, ZnO–ENPs caused a remarkable decrease in the physical properties of both soils, including BET surface area and pore volume. The reduction in both parameters in both soils is related to the low particle size resulting from the blocking of soil pores by ENPs [47] and preventing gaseous N_2_ from reaching them. Therefore, the application of 1% ZnO–ENPs demonstrated a notable effect on the physicochemical properties of both soils (Table 2), which play an important role in the Pi adsorption–desorption phenomenon.

### 3.2. Adsorption–Desorption Studies

#### 3.2.1. Pi Adsorption Kinetics

**Figure 1:**
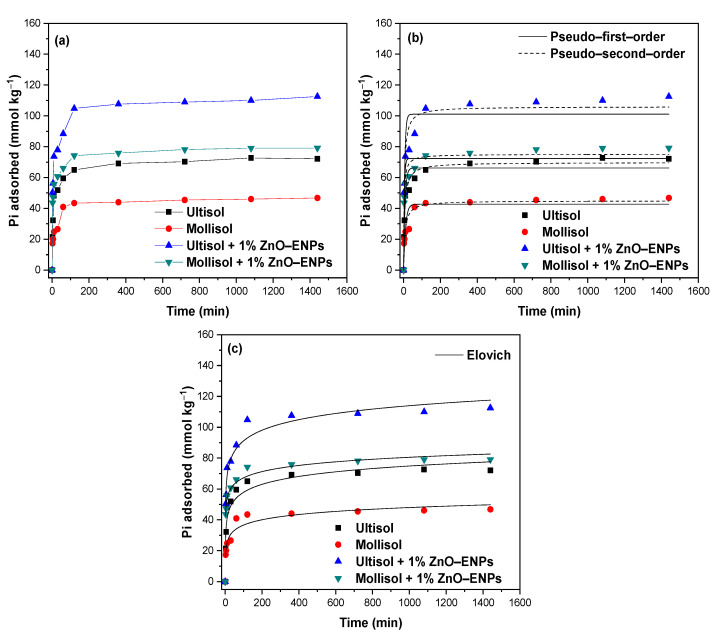
(**a**) Inorganic phosphorus (Pi) adsorption kinetics at pH 5.5 ± 0.1 of the solution in the presence of 1% ZnO–ENPs on Ultisol, and Mollisol soils, modelled by (**b**) pseudo-first-order (PFO) and pseudo-second-order (PSO), and (**c**) Elovich models.

The graphs of the Pi adsorption kinetics of the Ultisol, Mollisol, Ultisol + 1% ZnO–ENP, and Mollisol + 1% ZnO–ENP systems are shown in Figure 1a.

An increase in the amount of adsorbed Pi with contact time and the presence of 1% ZnO–ENPs was evident in both soils. In particular, the Pi adsorption kinetics followed a fast stage and a slow stage. The first fast stage was chemical adsorption [46] and occurred for the first 60 min, where Pi adsorption for Ultisol, Mollisol, Ultisol + 1% ZnO–ENPs, and Mollisol + 1% ZnO–ENPs reached 59.50 mmol kg^−1^ (22.74%), 40.89 mmol kg^−1^ (15.57%), 88.41 mmol kg^−1^ (34.37%), and 66.13 mmol kg^−1^ (25.59%), respectively. Conversely, the second slow stage was associated with a predominance of physical adsorption mechanisms [46] and started after 60 min. In particular, at 120 min Pi adsorption reached 64.97 mmol kg^−1^ (24.92%), 43.41 mmol kg^−1^ (16.59%), 104.81 mmol kg^−1^ (40.22%), and 74.21 mmol kg^−1^ (28.36%) for the Ultisol, Mollisol, Ultisol + 1% ZnO–ENP and Mollisol + 1% ZnO–ENP systems, respectively. This behavior can be explained by high concentrations of Pi rapidly saturating the available adsorption sites on the soil surface [46,47]. The capacity of Pi adsorbed by the different systems at 120 min remained practically constant up to 1440 min. The experimental data were modeled using the PFO, PSO, and Elovich models (Figure 1b and c), and values are provided in Table 3. 

According to the PSO model, the initial kinetic rate (h) had the following sequence: Mollisol + 1% ZnO–ENPs (28.15 kg mmol^−1^ min^−1^) > Ultisol + 1% ZnO–ENPs (25.14 kg mmol^−1^ min^−1^) > Ultisol (11.74 kg mmol^−1^ min^−1^) > Mollisol (6.07 kg mmol^−1^ min^−1^). This increase showed that the ZnO–ENPs at t→0 are forming new functional groups (≡Zn–OH and ≡Zn–OH_2_^+^) to the soil surface, and these had an affinity for Pi [48]. Similar results concerning the value of h were obtained by Manquián-Cerda et al. [49] for the adsorption of Cd^2+^ on soils in the presence of Ag–ENPs and Fe–ENPs.

The Elovich model describes the adsorption of an adsorbate on a heterogeneous surface through chemisorption [50]. According to the minimal differences shown in the values of r^2^ (i.e., 0.03) and χ^2^ (i.e., 29) (Table 3) by the PSO and Elovich models, it is possible that both models can adequately describe the Pi adsorption kinetic data on the Ultisol, Mollisol, Ultisol + 1% ZnO–ENPs, and Mollisol + 1% ZnO–ENPs systems. In particular, the order shown by the different systems with respect to ≈1 was as follows: Mollisol + 1% ZnO–ENPs > Ultisol + 1% ZnO–ENPs > Ultisol > Mollisol. This trend suggests that the presence of 1% ENPs makes the soil surface more heterogeneous. Moreover, this model may be used to extract the initial adsorption rate (α), which exhibited the following sequence: Mollisol + 1% ZnO–ENPs (6715.18 mmol kg^−1^ min^−1^) > Ultisol + 1% ZnO–ENPs (1022.03 mmol kg−1 min^−1^) > Ultisol (187.74 mmol kg^−1^ min^−1^) > Mollisol (85.01 mmol kg^−1^ min^−1^). This parameter aligns with the trend observed for the h value of the PSO model, which is attributable to its statistical consistency.

#### 3.2.2. Pi Adsorption Isotherms

**Figure 2:**
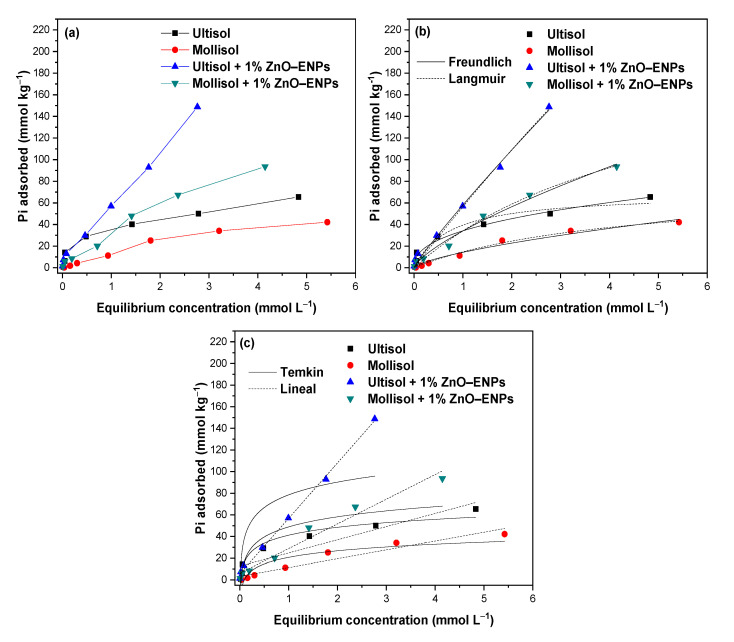
(**a**) Inorganic phosphorus (Pi) adsorption isotherms at pH 5.5 ± 0.1 in absence and presence of 1% ZnO–ENPs on Ultisol and Mollisol soils; (**b**) Pi adsorption isotherms in absence and presence of 1% ZnO–ENPs on Ultisol and Mollisol soils modelled by Langmuir and Freundlich and (**c**) Temkin and Lineal models.

The Pi adsorption isotherms in the Mollisol and Ultisol soils with and without 1% ZnO–ENPs are shown in Figure 2a.

It can be seen that the amount of Pi adsorbed by Ultisol, Mollisol, Ultisol + 1% ZnO–ENP, and Mollisol + 1% ZnO–ENP systems increases with the initial Pi concentration. The increase in the quantity of Pi adsorbed across various systems with the initial Pi concentration is due to the abundant availability of adsorption sites on the surfaces conducive to Pi molecule adsorption [50]. In particular, the experimental data of Pi adsorption isotherms for the Ultisol and Mollisol soils described S-type curves [51]. This is evidence that there is a stronger attraction between Pi molecules relative to the attraction of Pi to adsorption sites on the soil surface. Meanwhile, for the Ultisol + 1% ZnO–ENP and Mollisol + 1% ZnO–ENP systems, the Pi isotherm data described C-type curves [51], indicating a constant proportional affinity with the increase in the initial concentration of Pi molecules for soils + 1% ZnO–ENPs [51,52].

In the adsorption isotherms (Figure 2a), the experimental adsorption capacity (q_e_) of Pi is higher for the Ultisol soil than for the Mollisol soil. This difference in Pi q_e_ between the two soils could be associated with properties such as mineralogy, greater BET surface area, fewer negative ZP values (Figure S1), and more acidic pH of the Ultisol soil compared to the Mollisol soil (Table 2) [53]. This gives the Ultisol soil greater reactivity to adsorb more Pi molecules, whereas, in the presence of 1% ENPs, an increase in the concentration of Pi adsorbed on both soils was noted.

Experimental data were fitted to the linear, Langmuir, Freundlich, and Temkin models (Figure 2b,c and Table 4).

The Freundlich model best described the experimental data of Pi adsorption isotherms on the Ultisol soil (r^2^ = 0.976, and χ^2^ = 16), while the Langmuir model was a better fit to the Pi adsorption data by Mollisol soil (r^2^ = 0.991, and χ^2^ = 3). This showed that Pi adsorption occurs in multilayers on a heterogeneous surface for the Ultisol soil. In contrast, Pi adsorption on the Mollisol soil was in monolayers and on a homogeneous surface.

In the presence of 1% ZnO–ENPs, the Langmuir and Freundlich model showed minimal differences in r^2^ (r^2^ = 0.004) and χ^2^ (χ^2^ = 6) values (Table 4), which shows that both models can simultaneously describe the adsorption of Pi on the surface of soils + 1% ENPs. Likewise, the linear model showed values of r^2^ ≥ 0.970 for both soils with ENPs. This indicates that integrating ZnO–ENPs into soils not only creates additional adsorption sites due to their high surface area (10–25 m^2^ g^−1^) but also facilitates new mechanisms, as various models can describe how Pi could be adsorbed on the surfaces of these soils in the presence of 1% ZnO–ENPs [54]. For example, in addition to interacting directly with the soil’s inorganic and organic components, the linear model shows that between the Pi and the Ultisol + 1% ZnO–ENPs and Mollisol + 1% ZnO–ENPs, electrostatic interactions, Van der Waals interactions, and hydrophobic interactions could be occurring [54]. Furthermore, according to Luo et al. [30], the Pi present in the solution can be adsorbed on ZnO–ENPs through a Lewis acid–base interaction. On the other hand, under the conditions in which the Pi adsorption isotherm studies were conducted (pH 5.5), ZnO–ENPs can form an amorphous Zn(OH)_2_ coating, and PO_4_^3−^ can be adsorbed onto the hydroxylation surface via exchange with OH^−^, leading to the formation of an amorphous Zn phosphate layer [28]. At the same time, the surface of ZnO–ENPs was prone to being directly attacked by protons, resulting in a release of Zn^2+^ into the solution [44]. Concerning this, Wu et al. [55] reported that ZnO–ENPs at pH 5.5 dissolve easily, forming solvated ionic species of Zn^2+^_(aq)_ and Zn(OH)^+^_(aq)_. Once released from ZnO–ENPs in the soil solution, Zn^2+^ ions could bind to OM, clay minerals, and Fe/Al/Mn oxides (oxyhydrogen). Another possible process is due to the K_sp_ for zinc phosphate (Zn_3_(PO_4_)_2_ ) is extremely low (K_sp_ = 9.1 × 10^−33^) and when Zn^2+^ and Pi coexist in an acid solution, they are prone to likely to form a ZnO−Zn_3_(PO_4_)_2_ core−shell structure or/and a solid phase of Zn_3_(PO_4_)_2_·4H_2_O (hopeite) [29,56].

According to the Langmuir and Freundlich models (Table 4), the calculated adsorption energy (K_L_) and adsorption intensity (n), respectively, were notably lower for the Ultisol + 1% ZnO–ENP system (K_L_ = 0.04 and n = 1.10) compared to the Ultisol soil (K_L_ = 1.35, and n = 2.44). In contrast, the Mollisol + 1% ZnO–ENP system had a lower K_L_ value (K_L_ = 0.19) than the Mollisol soil (K_L_ = 0.24). A similar trend was reported by Mayakaduwa et al. [6], who determined the lowest adsorption energy for the soil with the highest Pi adsorption. This behavior in the K_L_ and n values was also observed by Mohebian et al. [57] for Cd^2+^ and Ni^2+^ adsorption on soils in the presence of ZnO–ENPs and CuO–ENPs.

#### 3.2.3. ENPs Doses

**Figure 3:**
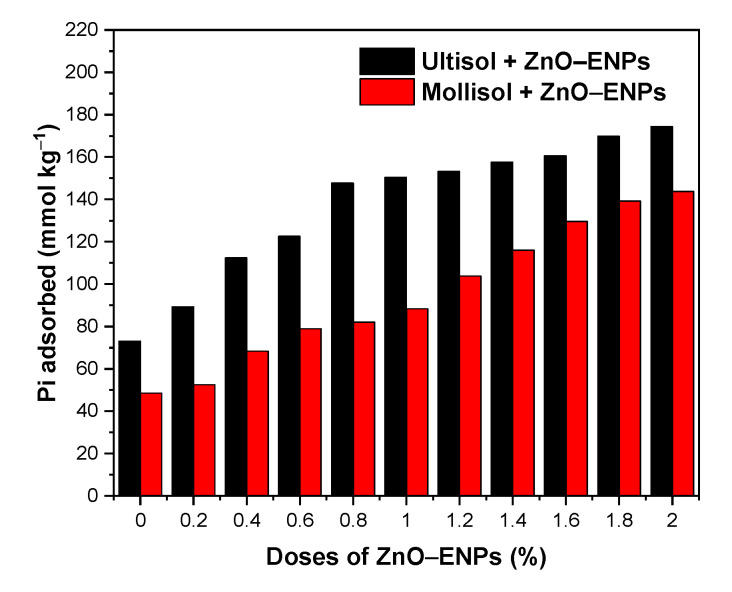
Weight effect of ZnO–ENPs on the inorganic phosphorus (Pi) adsorption on Ultisol and Mollisol soils.

Figure 3 shows the effect of an increasing dose of ZnO–ENPs from 0.2 to 2.0% in Pi adsorption on Mollisol and Ultisol soils.

The Ultisol soil without ENPs exhibited a Pi adsorption of 73.08 mmol kg^−1^ (28.23%), while the Mollisol soil without ENPs was 48.48 mmol kg^−1^ (18.65%). When the ZnO–ENP dose increased from 0.2% to 2%, the Pi adsorption in the Ultisol soil rose from 89.30 mmol kg^−1^ (34.73%) to 174.42 mmol kg^−1^ (62.54%), representing a 1.95–fold increase. In the Mollisol soil, the Pi adsorption increased from 52.46 mmol kg^−1^ (20.11%) to 143.80 mmol kg^−1^ (54.86%), indicating a 2.74–fold increase. Although increasing doses of ZnO–ENPs resulted in enhanced Pi adsorption in both soil types, this effect was significantly more pronounced in the Mollisol than the Ultisol. This effect is related to the incorporation of new available adsorption sites. Similar behaviors were observed by Suazo-Hernández et al. [58] for the adsorption of Pi onto volcanic soil with increasing doses of Cu–ENPs.

#### 3.2.4. pH Studies Regarding the Adsorption of Pi

**Figure 4:**
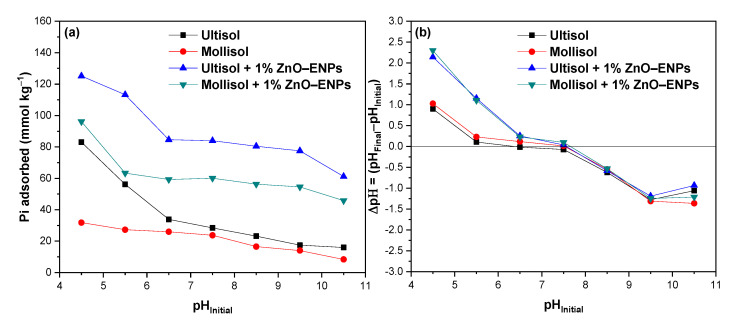
(**a**) pH_Initial_ effect of solution on inorganic phosphorus (Pi) adsorption on Ultisol and Mollisol soils without and with 1% ZnO–ENPs, and (**b**) variation of pH (ΔpH) before and after adsorption at different initial pH values.

The graph in Figure 4a shows the effect of the solution pH between 4.5 and 10.5 on Pi adsorption in the Ultisol and Mollisol soils without and with 1% ZnO–ENPs.

At an acidic pH of 4.5, significant Pi adsorption is observed in the Ultisol, Mollisol, Ultisol + 1% ZnO–ENP, and Mollisol + 1% ZnO–ENP systems, with values of 83.04 mmol kg^−1^ (32.15%), 31.82 mmol kg^−1^ (12.30%), 125.14 mmol kg^−1^ (48.30%), and 96.13 mmol kg^−1^ (37.21%), respectively. At a basic pH of 10.5, there is a marked decrease of Pi adsorbed of 15.97 mmol kg^−1^ (6.18%), 8.36 mmol kg^−1^ (3.23%), 61.20 mmol kg^−1^ (23.59%), and 45.84 mmol kg^−1^ (17.74%) for the Ultisol, Mollisol, Ultisol + 1% ZnO–ENP, and Mollisol + 1% ZnO–ENP systems, respectively. Therefore, in the presence of 1% ZnO–ENPs, regardless of the pH value, Pi adsorption in both soils increases.

The significant Pi adsorption at acidic pH levels across the several systems studied is due to the formation of inner-sphere complexes and electrostatic attraction. In contrast, at alkaline pH values, Pi adsorption decreased due to a decrease in inner sphere complexes and an increase in electrostatic repulsion. Several authors have reported this behavior for volcanic soils [58,59]. It has also been reported that ZnO–ENPs at basic pH exhibit a reduction in their dissolution rate, and there is a predominance of a negative surface charge [60], as well as for the adsorption of Pi by ENPs.

The results of this study were complemented by pH variations following Pi adsorption, the results of which are shown in Figure 4b. This was because the release of OH^−^ or H+ has been used to improve the understanding of the mechanism involved in Pi adsorption [58]. Figure 4b illustrates that when Pi adsorption occurs at pH 5.5, the final pH of the solution is around 5.7 for both soils, while in the presence of ENPs, a drastic increase in pH is observed, reaching values of 6.7. This difference may be related to the release of OH^−^ groups, mainly from the ENPs due to the ligand exchange between Pi and the OH^−^ groups on the surface of the ZnO–ENPs [30]. As a result, these findings could suggest that the interaction is more stable for 1% ENPs + soil systems than for soils alone.

#### 3.2.5. Pi Desorption Studies

**Figure 5:**
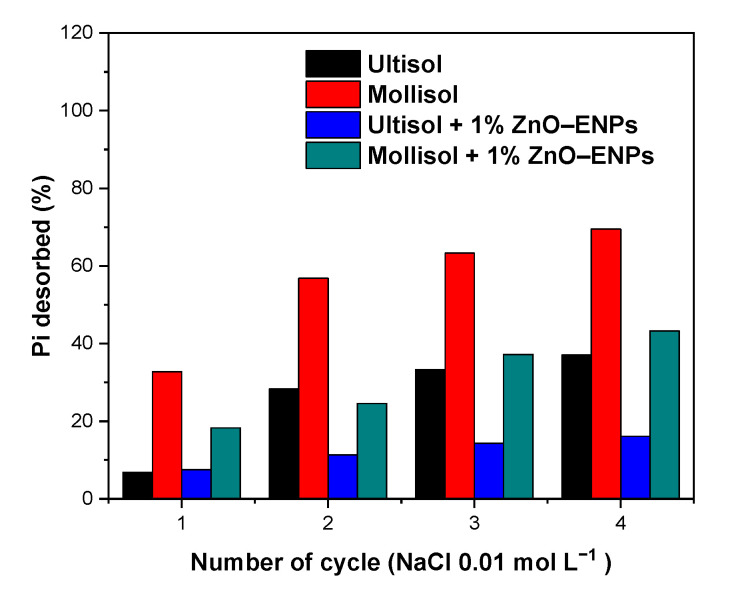
Inorganic phosphorus (Pi) desorption percentage obtained from Ultisol and Mollisol soils without and with 1% ZnO–ENPs.

Studies of Pi desorption from soil make it possible to predict the bioavailability of adsorbed Pi and its potential leaching into groundwater courses [61], and in this case, to evaluate the effect of 1% ZnO–ENPs on the strength of the Pi–soil interaction. In this study, Pi from the different systems was desorbed using NaCl 0.01 mol L^−1^ at pH 5.5. Figure 5 showed that after cycles, the desorption of Pi from the Mollisol, Mollisol + 1% ZnO–ENP, Ultisol, and Ultisol + 1% ZnO–ENP systems reached the following values: 69.51%, 43.24%, 37.05%, and 16.09%, respectively.

Therefore, after four cycles, Pi desorption was 1.87 higher in the Mollisol than in the Ultisol and was related to the values of n and K_L_ obtained from the Freundlich and Langmuir models (Table 4), respectively. This behavior could be associated with the differences in the physicochemical properties of both soils since Mollisol soil has a mineralogy dominated by montmorillonite [53], which has a low affinity for P, unlike Ultisol soil, which is mainly dominated by halloysite and kaolinite [62]. Furthermore, 1% ZnO–ENPs reduced Pi desorption by 0.62 and 0.43 times for Mollisol and Ultisol soils, respectively, compared to those without ENPs. Therefore, the ENPs are increasing the interaction strength between the surface of both soils and Pi, with this effect being much more pronounced for Ultisol soil. This is because Zn^2+^ cations can be adsorbed in greater amounts onto Ultisol than onto Mollisol soil; hence, the Zn^2+^ released from the ENPs into the solution could have been retained by Ultisol soil, as opposed to the low capacity of Mollisol soil to retain Zn^2+^ cations [63].

## 4. Conclusions

Characterization of Ultisol and Mollisol soils showed that 1% ZnO–ENPs generated a variation in physicochemical properties. The pseudo-second-order and Elovich models indicated that the Pi was adsorbed on a heterogeneous surface through chemisorption. The adsorption isotherms of inorganic phosphorus (Pi) on Ultisol and Mollisol soils were described using the Freundlich and Langmuir models, respectively. Meanwhile, in the presence of 1% ZnO–ENPs, the linear, Langmuir, and Freundlich models (r^2^ ≥ 0.928 and χ^2^ ≤ 36) showed a good fit, which accounts for the different mechanisms involved in Pi adsorption in soil + ENP systems. The percentage of Pi desorption decreased with ENPs, suggesting a higher stability of Pi binding to the soil surface. Finally, ENPs may be an alternative material for agricultural soils that receive high doses of phosphate fertilizers, as its application can contribute to preventing Pi mobility and the contamination of nearby water bodies.

## Figures and Tables

**Table 1:** Parameters evaluated in inorganic phosphorus adsorption experiments.

Parameters	Weight ENPs	pH	Kinetics	Isotherms
Agitation time (min)	1440	1440	0–1440	1440
ENPs doses (g)	0.001–0.010	0.005	0.005	0.005
Adsorbent dose (g)	0.5	0.5	0.5	0.5
Initial P concentration (mmol L^−1^)	6.46	6.46	6.46	0.02–6.46
pH solution	5.5	4.5–10.5	5.5	5.5
Volume of adsorbate (mL)	20	20	20	20
Shaking speed (rpm)	200	200	200	200

**Table 2:** Physicochemical properties of Ultisol and Mollisol soils without and with 1% ZnO–ENPs.

Soil Analysis	Ultisol	Mollisol	Ultisol + 1% ZnO–ENPs	Mollisol + 1% ZnO–ENPs
Available N (mg kg^−1^)	10	10	9	8
Available P (mg kg^−1^)	11	37	4	15
pH_H2O_	6.14	6.33	8.08	8.14
Electrical conductivity (dS m^−1^)	0.039	0.078	0.054	0.097
Organic matter (%)	2.07	2.36	2.13	2.68
K (cmol+ kg^−1^)	0.21	0.80	0.20	0.73
Na (cmol+ kg^−1^)	0.11	0.11	0.10	0.09
Ca (cmol+ kg^−1^)	6.98	7.98	7.08	7.96
Mg (cmol+ kg^−1^)	2.49	1.77	2.46	1.71
ECEC (cmol+ kg^−1^) *	9.82	10.68	9.87	10.51
Zn–DTPA (mg kg^−1^)	0.69	15.3	871	912
Cu–DTPA (mg kg^−1^)	2.09	1.64	0.25	0.24
Fe–DTPA (mg kg^−1^)	57.0	34.0	1.56	0.51
BET surface area (m^2^ g^−1^)	25.490	22.998	20.685	16.279
Average pore volume (cm^3^ g^−1^)	0.025	0.027	0.021	0.018
Average pore diameter (nm)	3.827	3.827	3.784	3.827

* Effective cation exchange capacity.

**Table 3:** Pseudo-first-order, pseudo-second-order, and Elovich parameters obtained from inorganic phosphorus adsorption kinetics at pH 5.5 ± 0.1 in the without and with of 1% ZnO–ENPs on Ultisol and Mollisol soils.

Kinetic Parameters	Ultisol	Mollisol	Ultisol + 1% ZnO–ENPs	Mollisol + 1% ZnO–ENPs
q_exp_ (mmol kg^−1^)	72.10 ± 13.27	46.80 ± 11.06	112.53 ± 14.12	79.14 ± 10.05
q_exp_ (%)	27.73	17.89	42.98	30.58
	Pseudo–first–order			
q_e_ (mmol kg^−1^)	66.25 ± 2.32	42.75 ± 2.40	101.08 ± 4.57	72.35 ± 2.90
k_1_ (×10^−3^ min^−1^)	128.71 ± 22.97	93.51 ± 26.23	171.75 ± 40.10	241.90 ± 53.01
r^2^	0.943	0.870	0.892	0.902
χ^2^	36	35	146	61
	Pseudo–second–order			
q_e_ (mmol kg^−1^)	69.81 ± 1.44	44.98 ± 1.77	105.95 ± 3.32	75.11 ± 2.01
k_2_ (×10^−3^ kg mmol^−1^ min^−1^)	2.41 ± 0.00	3.00 ± 0.00	2.24 ± 0.00	4.99 ± 1.05
h (mmol kg^−1^ min^−1^)	11.74 ± 0.00	6.07 ± 0.00	25.14 ± 0.00	28.15 ± 0.00
r^2^	0.983	0.942	0.954	0.961
χ^2^	11	16	61	24
	Elovich			
α (mmol kg^−1^ min^−1^)	187.74 ± 14.74	85.01 ± 6.31	1022.03 ± 70.86	6715.18 ± 118.24
β (kg mmol^−1^)	0.14 ± 0.01	0.20 ± 0.02	0.10 ± 0.00	0.173 ± 0.012
r^2^	0.953	0.951	0.976	0.987
χ^2^	30	13	32	8

**Table 4:** Langmuir, Freundlich, and Temkin parameters for inorganic phosphorus adsorption isotherms at pH 5.5 ± 0.1 without and with 1% ZnO–ENPs on Ultisol and Mollisol soils.

	Ultisol	Mollisol	Ultisol + 1% ZnO–ENPs	Mollisol + 1% ZnO–ENPs
	Langmuir			
K_L_	1.35 ± 0.60	0.24 ± 0.06	0.04 ± 0.00	0.19 ± 0.05
q_max_	68.66 ± 8.60	76.61 ± 10.11	1650.87 ± 127.74	214.89 ± 37.25
r^2^	0.947	0.991	0.992	0.992
χ^2^	36	3	29	11
	Freundlich			
K_F_	34.15 ± 2.00	14.25 ± 1.66	58.09 ± 3.06	1.37 ± 0.11
n	2.44 ± 0.27	1.48 ± 0.18	1.10 ± 008	34.07 ± 2.43
r^2^	0.976	0.972	0.993	0.988
χ^2^	16	9	23	17
	Temkin			
K_T_	48.90 ± 15.09	10.77 ± 5.09	100.60 ± 19.67	37.42 ± 33.18
B_T_	230.31 ± 20.62	279.44 ± 51.81	143.16 ± 44.87	180.69 ± 48.17
r^2^	0.962	0.855	0.673	0.740
χ^2^	26	50	1150	393
	Linear			
K (L kg^−1^)	12.15 ± 2.13	8.14 ± 1.02	51.27 ± 1.14	22.80 ± 1.82
r^2^	0.866	0.928	0.998	0.970

## Data Availability

Data will be made available on request.

## Supplementary Materials

The following supporting information can be downloaded at: https://www.mdpi.com/article/10.3390/toxics13050363/s1, Figure S1: Zeta potential for Ultisol and Mollisol soils. Table S1. Kinetic models used for description of inorganic phosphorus adsorption. Table S2. Isotherm models used for description of inorganic phosphorus adsorption. References [64,65,66] are cited in the supplementary materials.
