# Supplementary material for: Immobilization of Inorganic Phosphorus on Soils by Zinc Oxide Engineered Nanoparticles
Source: Toxics. 2025 Apr 30;13(5):363. doi: 10.3390/toxics13050363 (PMC12116105; doi:10.3390/toxics13050363)
Supplement: Supplementary file 1 [file toxics-13-00363-s001.zip › toxics-3553265-supplementary.pdf]

toxics

Supplementary information

Immobilization of inorganic phosphorus on soils by zinc oxide-engineered nanoparticles

Jonathan Suazo-Hernández [1,2*], Rawan Mlih [3,4], Marion Bustamante [5], Carmen Castro-Castillo [6], María de la Luz Mora [1,2], María de los Ángeles Sepúlveda-Parada [7], Catalina Mella [8], Pablo Cornejo [9,10], Antonieta Ruiz [2*]

1 Center of Plant, Soil Interaction and Natural Resources Biotechnology, Scientific and Biotechnological Bioresource Nucleus (BIOREN-UFRO), Universidad de La Frontera, Avenida Francisco Salazar, Temuco 01145, Chile; mariluz.mora@ufrontera.cl
2 Department of Chemical Sciences and Natural Resources, Universidad de La Frontera, Avenida Francisco Salazar, P.O. Box 54-D, Temuco 01145, Chile
3 Institute of Bio- and Geosciences, Agrosphere (IBG-3), Forschungszentrum Juelich (FZJ), 52425 Juelich, Germany; r.mlih@fz-juelich.de
4 Institute of Water and Environment (IWE), Al Azhar University-Gaza, Gaza P.O. Box. 1277, Palestine
5 Doctoral Program in Engineering at the MacroFacultad de Ingeniería UFRO-UBB-UTAL, Temuco 4780000, Chile; marionbustamante.v@gmail.com
6 LabMAM, Department of Chemical Engineering, Biotechnology and Materials, FCFM, Universidad de Chile, Santiago 8370456, Chile; carmen.castro.c@uchile.cl
7 Spectroscopy Laboratory (Vis-IF) and Sustainable Soil Management, Department of Soil Science and Natural Resources, Faculty of Agronomy, Universidad de Concepción, Vicente Méndez 595, Casilla 537, Chillán 3812120, Chile; angeles.sepulvedap@gmail.com (M.d.L.A-S)
8 Doctorado en Ciencias de Recursos Naturales, Universidad de La Frontera, Temuco 4811230, Chile; c.mella10@ufromail.cl
9 Plant Stress Physiology Laboratory, Centro de Estudios Avanzados en Fruticultura (CEAF), Rengo 2940000, Chile; pablo.cornejo@pucv.cl
10 Centro Tecnológico de Suelos y Cultivos (CTSyC), Facultad de Ciencias Agrarias, Universidad de Talca, Talca 3460000, Chile
* Correspondence : jonathan.suazo@ufrontera.cl (J.S.-H.); maria.ruiz@ufrontera.cl (A.R.)

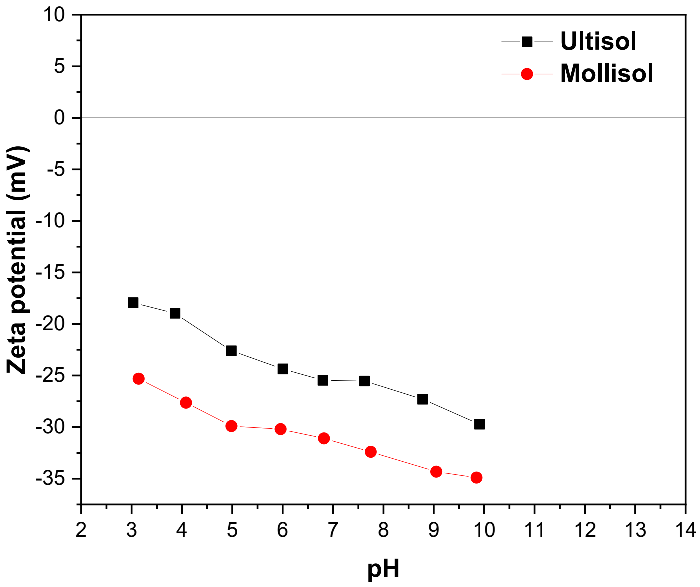

Fig S1: Zeta potential for Ultisol and Mollisol soils.

Table S1. The kinetic models used for the description of inorganic phosphorus adsorption.

Kinetic Equations	Expression Formula	Parameters	References
Pseudo–first–order (PFO)	$q_t = q_e(1 - e^{-k_1 t})$	q_t = Amount of anion adsorbed at any time (mmol kg^{-1}).	
Pseudo–second–order (PSO)*	$q_t = \frac{k_2 q_e^2 t}{1 + k_2 q_e t}$	q_e = Amount of anion adsorbed at equilibrium (mmol kg^{-1}). k_1 = PFO rate constant (min^{-1}). k_2 = PSO rate constant (kg mmol^{-1} min^{-1}). t = Time (min)	[40,64]
Elovich	$q_t = \frac{1}{\beta} \ln(1 + \alpha\beta t)$	α = Initial rate constant (mmol kg^{-1} min^{-1}). β = Number of sites available for the adsorption and desorption constant (kg mmol^{-1}).	

*From PSO initial adsorption rate (h), can be calculated by multiplying $k_2 q_e^2$ (mmol·kg^{-1}·min^{-1}).

Table S2. The isotherm models used for the description of inorganic phosphorus adsorption.

Isotherm Equations	Expression Formula	Parameters	References
Langmuir	$q_e = \frac{q_{max} K_L C_e}{1 + K_L C_e}$	q_e = Amount of adsorbed anion per unit mass of the adsorbent at equilibrium (mmol kg^{-1}).	
Freundlich	$q_e = K_F C_e^{1/n}$	q_{max} = Maximum adsorption capacity (mmol kg^{-1}). C_e = concentration of anion at equilibrium in the solution (mmol L^{-1}). K_L = Constant related to the affinity (L mmol^{-1}). K_F = Freundlich adsorption coefficient (mmol kg^{-1}) (L mmol^{-1})$^{1/n}$. n = Adsorption intensity (1 < n < 10).	[54,65,66]
Temkin	$q_e = \frac{RT}{b} \ln(AC_e)$	b = Temkin constant related to the heat of adsorption (J mol^{-1}). A = Temkin isotherm constant (L g^{-1}). R = Universal gas constant (8.314 J mol^{-1}K^{-1}). T = Absolute temperature (K).	
Lineal	$q_e = K_H C_e$	K_H = Partition coefficient (L·g^{-1}).	

References

64. Huang, Z.; Li, Y.; Chen, W.; Shi, J.; Zhang, N.; Wang, X.; Li, Z.; Gao, L.; Zhang, Y. Modified Bentonite Adsorption of Organic Pollutants of Dye Wastewater. *Mater. Chem. Phys.* **2017**, *202*, 266–276, doi:10.1016/j.matchemphys.2017.09.028.

65. Suazo-Hernández, J.; Sepúlveda, P.; Manquián-Cerda, K.; Ramírez-Tagle, R.; Rubio, M.A.; Bolan, N.; Sarkar, B.; Arancibia-Miranda, N. Synthesis and Characterization of Zeolite-Based Composites Functionalized with Nanoscale Zero-Valent Iron for Removing Arsenic in the Presence of Selenium from Water. *J. Hazard. Mater.* **2019**, *373*, 810–819, doi:10.1016/j.jhazmat.2019.03.125.

66. Alkaim, A.F.; Sadik, Z.; Mahdi, D.K.; Alshrefi, S.M.; Al-Sammarraie, A.M.; Alamgir, F.M.; Singh, P.M.; Aljeboree, A.M. Preparation, Structure and Adsorption Properties of Synthesized Multiwall Carbon Nanotubes for Highly Effective Removal of Maxilon Blue Dye. *Korean J. Chem. Eng.* **2015**, *32*, 2456–2462, doi:10.1007/s11814-015-0078-y.
